# Can Contrast-Enhanced Ultrasound Reduce the False-Negative Rate of Metastatic Axillary Lymph Nodes After Neoadjuvant Therapy in Breast Cancer?

**DOI:** 10.3390/cancers18172744

**Published:** 2026-08-24

**Authors:** Sijia Zhao, Jia Luo, Manying Li, Jiaping Li, Jiaqian Zhong, Peiwei Chen, Xiaoyan Xie, Ying Lin, Yanling Zheng

**Affiliations:** 1 Department of Ultrasound, The First Affiliated Hospital, Sun Yat-sen University, Guangzhou 510080, China; 2 Department of Breast Surgery, The First Affiliated Hospital, Sun Yat-sen University, Guangzhou 510080, China

**Keywords:** false-negative rate, contrast-enhanced ultrasound, breast cancer, lymph nodes, neoadjuvant therapy

## Abstract

Background: Accurate assessment of axillary lymph node (ALN) status after neoadjuvant therapy (NAT) remains essential for surgical planning in patients with initially node-positive breast cancer. This study evaluated the potential value of contrast-enhanced ultrasound (CEUS) in conjunction with sentinel lymph node biopsy (SLNB) for post-NAT axillary assessment and explored clinicopathologic factors associated with lymph node (LN) status. Methods: In this retrospective study, 179 patients with cytologically confirmed node-positive breast cancer underwent CEUS after NAT, followed by surgical pathological evaluation. The observed false-negative rate (FNR) of post-NAT CEUS combined with SLNB was compared with that of SLNB alone and post-NAT ultrasound (US) combined with SLNB. Univariable and multivariable generalized estimating equation (GEE) analyses were performed to identify factors associated with LN-level outcomes. Results: A total of 213 sentinel lymph nodes were analyzed. CEUS combined with SLNB had an observed FNR of 13.25% (95% CI, 7.56–22.19%), which was numerically lower than the FNRs of SLNB alone and US combined with SLNB. HER2 status, hormone receptor status, breast pathological response after NAT, and CEUS findings were significantly associated with nodal status in the univariable GEE model. Exploratory multivariable GEE analyses identified pre-NAT HER2 positivity as an independent protective factor for false-negative LN outcomes, whereas pre-NAT ER status and residual invasive carcinoma were independently associated with CEUS-negative/SLNB-positive nodal status. The exploratory models showed high apparent discrimination, with AUCs of 0.941 (95% CI, 0.890–0.991) and 0.917 (95% CI, 0.866–0.968). Conclusions: In this single-center retrospective cohort, the addition of post-NAT CEUS to SLNB was associated with a lower observed FNR than SLNB alone. These findings support the potential role of CEUS as a complementary tool for post-NAT axillary assessment but require prospective studies with external validation.

## Simple Summary

Patients with breast cancer who receive neoadjuvant therapy require accurate evaluation of axillary lymph nodes to guide surgical treatment. However, sentinel lymph node biopsy may fail to identify residual nodal disease in some patients. We investigated whether contrast-enhanced ultrasound performed before surgery could provide additional information for post-neoadjuvant axillary assessment and explored clinical factors associated with lymph node status. We found that combining contrast-enhanced ultrasound with sentinel lymph node biopsy was associated with a lower false-negative rate than sentinel lymph node biopsy alone and identified tumor characteristics associated with residual nodal disease. These findings suggest that contrast-enhanced ultrasound may have potential as a complementary tool for post-neoadjuvant axillary assessment, although its clinical value requires confirmation in larger prospective studies with external validation.

## 1. Introduction

Breast Cancer is the most frequently diagnosed malignancy among women worldwide, with an incidence more than twice that of lung cancer, which is the second most common malignancy, and its global incidence has continued to increase over the past decade [1]. Accurate assessment of axillary lymph node (ALN) metastasis is critical for disease staging and therapeutic selection in breast cancer [2,3]. Sentinel lymph nodes (SLNs), defined as the first draining lymph nodes (LNs) receiving lymphatic drainage from the primary tumor and therefore the first sites to which metastatic cells may spread, serve as reliable indicators for the detection of regional LN metastases, including micrometastatic disease [4]. Neoadjuvant therapy (NAT) has become a standard treatment approach for patients with LN-positive breast cancer because it may downstage axillary disease and reduce the extent of axillary surgery, thereby decreasing treatment-related morbidity [5,6]. Following NAT, axillary pathological complete response (pCR) is achieved in up to 70% of patients with initially node-positive disease [5,6]. After these favorable responses, most patients continue to undergo axillary lymph node dissection (ALND) or sentinel lymph node biopsy (SLNB). However, ALND may result in lymphedema and injury to surrounding structures, whereas SLNB after NAT remains limited by a relatively high false-negative rate (FNR) [7,8,9,10,11]. Recent efforts have focused on reducing the FNR of SLNB while minimizing surgical morbidity. Although dual-tracer SLNB using radioisotopes and blue dye has demonstrated acceptable FNRs in patients with node-positive breast cancer, logistical and regulatory challenges related to radioisotope use have limited its widespread implementation [12]. In this context, preoperative adjunctive imaging techniques may provide an alternative strategy for improving SLN identification and reducing the FNR of SLNB.

Ultrasound (US) remains the primary imaging modality for post-NAT evaluation of ALNs in patients with breast cancer [13]. However, conventional US has limited diagnostic performance for ALN assessment because of its relatively low accuracy in differentiating benign nodes from nodes with residual metastatic disease. In addition, treatment-related fibrosis and tumor regression following NAT further complicate the morphologic evaluation of ALNs [14,15,16]. Contrast-enhanced ultrasound (CEUS) has emerged as a promising imaging technique for lymphatic mapping and nodal assessment. Following subcutaneous or intravenous administration of microbubble contrast agents, CEUS enables real-time visualization of lymphatic channels and draining LNs through enhancement of lymphatic and capillary flow [17]. Percutaneous CEUS (PCEUS) is particularly valuable for SLN mapping, allowing accurate localization and evaluation of nodal status [18]. In breast cancer, the SLN is defined as the first draining LN reached by contrast-agent microbubbles [19]. Compared with conventional US, CEUS provides additional functional and morphologic information, potentially improving the visualization and characterization of SLNs and facilitating preoperative assessment to guide surgical decision-making. Previous studies have further optimized CEUS-based LN classification systems to improve diagnostic performance in patients both before and after NAT [17,18].

To our knowledge, no prior study has comprehensively evaluated the FNR of SLNB with the adjunctive use of CEUS in patients with initially node-positive breast cancer after NAT. Therefore, we analyzed clinicopathologic characteristics and CEUS imaging features in patients with initially LN-positive breast cancer treated with NAT to determine whether CEUS could reduce the FNR of SLNB. Furthermore, univariable and multivariable GEE analyses were performed to identify factors associated with LN-level outcomes, and receiver operating characteristic (ROC) curve analysis was used to evaluate the discriminative performance of the exploratory models.

## 2. Materials and Methods

### 2.1. Patients

**Figure 1:**
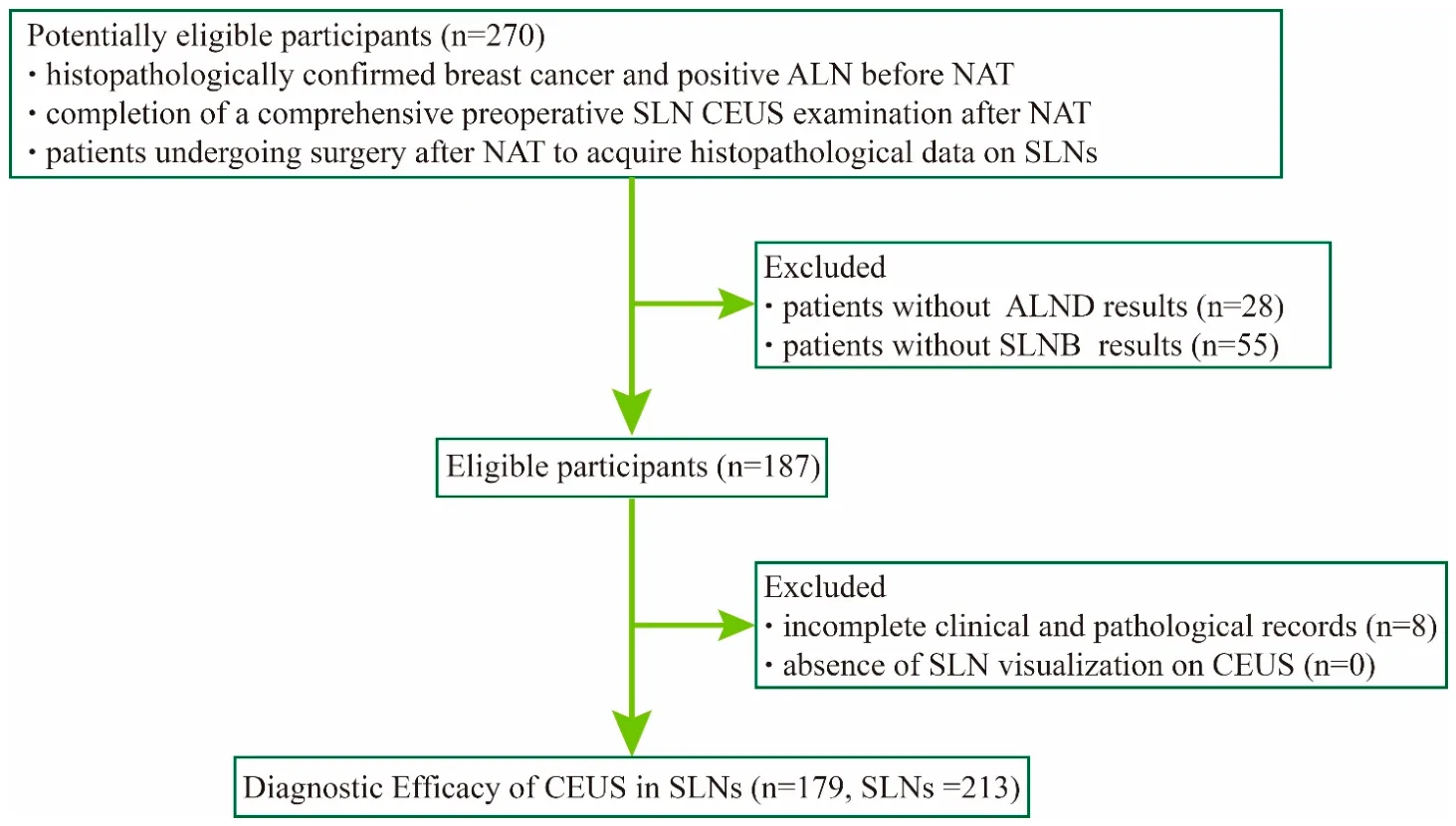
Flowchart of study subjects. ALN, axillary lymph node; NAT, neoadjuvant therapy; SLN, sentinel lymph node; CEUS, contrast-enhanced ultrasound; ALND, axillary lymph node dissection; SLNB, sentinel lymph node biopsy.

A total of 270 consecutive female patients with breast cancer treated between September 2019 and March 2026 were retrospectively enrolled from the First Affiliated Hospital of Sun Yat-sen University. Owing to the retrospective nature of the study, the requirement for written informed consent was waived. The eligibility criteria were listed below: (a) pathologically confirmed breast cancer with cytologically or pathologically confirmed ALN metastasis before NAT; (b) completion of comprehensive preoperative ALN evaluation with CEUS after NAT, including both percutaneous and intravenous CEUS (PCEUS and IVCEUS); (c) subsequent surgical treatment with available histopathologic results of LNs. The following exclusion criteria were applied: (a) absence of SLNB or ALND results; (b) incomplete clinicopathologic data; (c) inadequate US image quality; (d) failure of SLN visualization on CEUS. Ultimately, a total of 179 patients with 213 SLNs were eligible for final analysis (Figure 1). All analyses involving SLNs were performed at the lymph-node level. The study protocol was performed in accordance with the Declaration of Helsinki (revised 2020) and received approval from the Ethics Committee of the First Affiliated Hospital of Sun Yat-sen University [(2020)316].

### 2.2. Instruments and Methods

Preoperative breast and axillary ultrasonography were conducted approximately two weeks before surgery using one of the following systems: Siemens ACUSON (Siemens Healthineers, Erlangen, Germany; equipped with a 10L4 probe), Mindray Resona 7 (Mindray, Shenzhen, China; equipped with an L9-3 probe), Mindray Resona 9 (equipped with an L9-3U probe), or Canon Aplio1900 (Canon Medical Systems, Tokyo, Japan; equipped with an 18LX5 probe). Imaging gain, mechanical index, and contrast-agent injection protocols were standardized across all four ultrasound platforms to minimize scanner-related variability. The US contrast agent consisted of a sulfur hexafluoride-filled microbubble suspension (SonoVue, Bracco, Italy), prepared by reconstituting lyophilized sulfur hexafluoride microbubbles (59 mg) in 5 mL of sterile saline, followed by agitation for 30 s to ensure homogeneous dispersion before administration.

With the patient in the supine position and the arms abducted, conventional breast and axillary ultrasonography was performed. After skin sterilization, 1.0 mL of contrast-agent suspension was administered into the upper outer periareolar region on the affected side to facilitate lymphatic enhancement. Dynamic enhancement of the SLN was continuously monitored in dual-image mode, and its cutaneous projection was marked. The probe was then positioned over the marked projection and switched to B-mode imaging to identify the largest longitudinal section of the SLN. Thereafter, 2.4 mL of microbubble contrast agent was administered intravenously with the transducer held stationary to evaluate nodal perfusion characteristics.

Subsequently, US features of the SLN were systematically recorded. SLN characterization was performed according to previously established criteria [18]. Specifically, P-CEUS enhancement patterns of SLNs were classified into six types. Type I showed partial cortical enhancement with nonenhancement of the remaining cortex and uneven cortical thickening; Type II, partial cortical filling defects; Type III, no enhancement; Type IV, homogeneous high enhancement; Type V, diffuse heterogeneous high enhancement; and Type VI, including VIa, nonenhancement/low enhancement of the lymphatic hilum with homogeneous high enhancement of the cortex; VIb, Type IV, V, or VIa enhancement in one half of the SLN with nonenhancement in the other half; and VIc, partial cortical enhancement with nonenhancement of the remaining cortex and uniform cortical thickening. Types I–III were classified as malignant, whereas Types IV–VI were classified as benign. IV-CEUS enhancement patterns were classified into four types: Type I, homogeneous high enhancement; Type II, diffuse heterogeneous high enhancement; Type III, nonenhancement/low enhancement of the lymphatic hilum with homogeneous high enhancement of the cortex; and Type IV, partial cortical filling defects, with the remaining cortex showing Type I, II, or III enhancement. The IV-CEUS enhancement sequence was classified according to the direction of microbubble movement as centrifugal, centripetal, or diffuse enhancement. An SLN was classified as malignant on IV-CEUS if any of the following was present: (I) centripetal enhancement; (II) diffuse enhancement; or (III) Type IV enhancement. An SLN was ultimately classified as malignant if it was classified as malignant on P-CEUS and/or IV-CEUS [18]. All US examinations were performed by two radiologists with more than 5 years of experience in conventional US and CEUS. The readers were blinded to the pathological results. CEUS nodal status was classified as positive or negative according to the predefined enhancement characteristics described above. In cases of discrepancy, final interpretation was reached by consensus after joint review.

A predefined composite diagnostic algorithm was established for the combined evaluation of imaging findings and SLNB results. Patients with either positive CEUS or positive SLNB were classified as positive and considered for ALND, whereas patients with both negative CEUS and negative SLNB were classified as negative and did not undergo ALND. Under this unified diagnostic pathway, the FNR of post-NAT CEUS combined with SLNB was compared with those of SLNB alone and post-NAT US combined with SLNB.

### 2.3. Pathological Evaluation

Pathologic specimens were obtained at the time of surgery following NAT. The pCR of primary breast tumor was defined as no residual invasive tumor within the breast, with or without residual ductal carcinoma in situ. ALN pCR was defined as the absence of residual metastatic disease in all examined ALNs.

Immunohistochemistry (IHC) was used to determine estrogen receptor (ER), progesterone receptor (PR), human epidermal growth factor receptor 2 (HER2), and Ki-67 status. Tumors with at least 1% nuclear staining were considered ER- or PR-positive [20]. HER2 positivity was defined as IHC 3+ or IHC 2+ with positive FISH findings [21]. The Ki-67 proliferation index was dichotomized into low- and high-proliferation groups using a 20% cutoff value [22,23].

### 2.4. Surgical Management

SLNB was generally performed 1 day after SLN-CEUS. After disinfection, 0.2–0.5 mL of methylene blue was injected intradermally into the upper outer quadrant of the areola, followed by 5–10 min of breast massage. SLNs were identified by tracing the blue-stained lymphatic vessels through an incision along the lateral border of the pectoralis major muscle and were subsequently submitted for histopathological examination. Pathologists were blinded to SLN-CEUS and other imaging findings.

### 2.5. Statistical Analysis

Statistical analysis was conducted with RStudio 4.4.3, SPSS 25.0, and GraphPad Prism 9.0. Continuous variables with a normal distribution are reported as mean ± standard deviation (SD) and were compared with the independent-samples t test. Median (interquartile range) values were reported for non-normally distributed continuous variables and compared using the Mann–Whitney U test. The χ^2^ test was used for categorical variables. Diagnostic performance was assessed using sensitivity, specificity, PPV, NPV, accuracy, FNR, and FPR, with 95% confidence intervals (CIs) calculated using exact binomial methods. For exploratory analyses of factors associated with LN pathological status, the SLN was used as the unit of analysis. Because individual patients could contribute multiple SLNs, univariable GEE logistic regression models were fitted to account for within-patient clustering, with patient ID specified as the clustering variable and an exchangeable working correlation structure. Odds ratios (ORs) and 95% CIs were reported. Given the limited number of outcome events, LASSO logistic regression was used for sparse-term selection. The selected terms were subsequently entered into a multivariable GEE logistic regression model, with patient ID specified as the clustering variable to account for within-patient correlation. The discriminative performance of the multivariable GEE models was evaluated using receiver operating characteristic (ROC) curve analysis and the area under the ROC curve (AUC). Two-sided *p* values less than 0.05 were considered significant.

For SLNB alone, a false-negative (FN) event was defined as a negative SLNB result in a patient with residual metastatic disease identified in ALND. For post-NAT US combined with SLNB, an FN event was defined as negative findings on both US and SLNB in patients despite residual nodal disease identified by ALND. For post-NAT CEUS plus SLNB, an FN event was defined similarly, based on negative CEUS and SLNB findings despite residual nodal disease identified by ALND. The FNR was derived from the ratio of FN cases to all cases with residual axillary disease confirmed pathologically (as detected at SLNB and/or ALND). True-negative (TN) status was defined as concordant negative findings on post-NAT CEUS, SLNB, and ALND.

## 3. Results

### 3.1. Participant Characteristics

Among the 213 SLNs analyzed, 144 were classified as CEUS-negative and 69 as CEUS-positive. Clinicopathologic characteristics were compared between the two SLN-level groups, comprising age, menopausal status, tumor size, tumor histology, clinical T stage, clinical N stage, pathologic response, type of surgery, and the number of SLNs and ALNs retrieved (Table 1). No significant differences were observed between groups for these variables, except for pre-NAT HER2 status (*p* = 0.001). Specifically, the CEUS-positive SLNs demonstrated a higher proportion of HER2-negative tumors, whereas HER2 positivity was more prevalent in the CEUS-negative SLNs.

### 3.2. FNR of SLNB Alone and SLNB Combined with Post-NAT US

Overall, the FNR associated with SLNB alone was 30.12% (95% CI: 21.31%, 40.69%), indicating that 25 cases with residual axillary nodal metastasis would have been misclassified as node negative if SLNB had been used as the sole axillary examining method (Figure S1A). In comparison, the FNR for post-NAT US-assisted SLNB decreased to 16.87% (95% CI: 10.32%, 26.34%). Under this combined strategy, only 14 ALNs with residual axillary disease would have been undertreated in the absence of subsequent ALND. The false-positive rate (FPR) of post-NAT US-assisted SLNB was 16.15% (95% CI: 10.82%, 23.44%) (Figure S1B).

### 3.3. FNR of Post-NAT CEUS-Assisted SLNB

**Figure 2:**
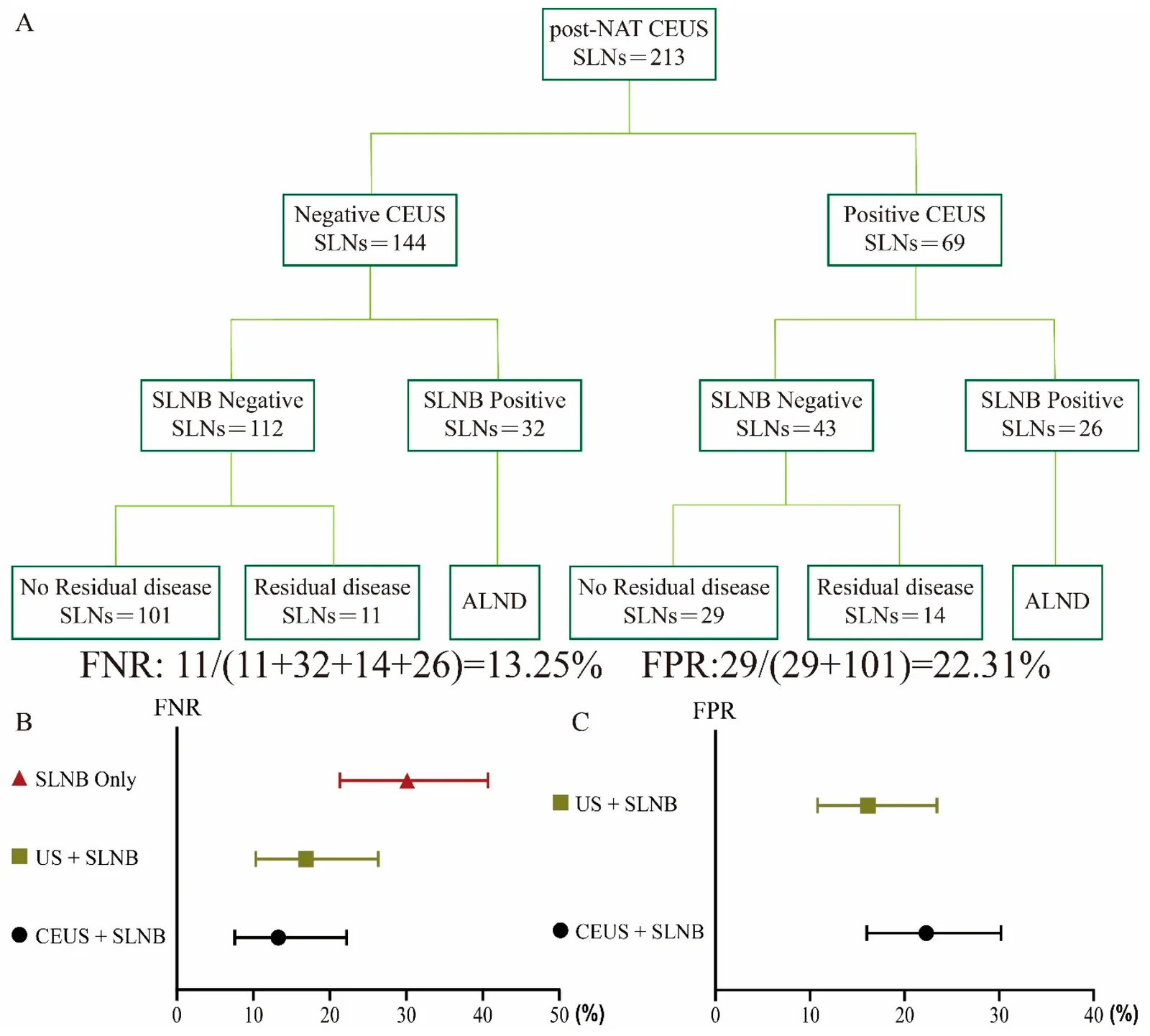
FNR and FPR of patients. (**A**) Patients undergoing SLNB based on post-NAT CEUS findings (SLNs = 11); (**B**) Comparison of FNR for patients of SLNB only, US plus SLNB, and CEUS plus SLNB; (**C**) Comparison of FPR for patients of US plus SLNB and CEUS plus SLNB. FNR, false-negative rate; FPR, false-positive rate; NAT, neoadjuvant therapy; CEUS, contrast-enhanced ultrasound; ALND, axillary lymph node dissection; SLNB, sentinel lymph node biopsy.

We further assessed the diagnostic performance of post-NAT CEUS combined with SLNB to assess whether CEUS could provide additional information for post-NAT axillary assessment. The FNR of combined post-NAT CEUS and SLNB was 13.25% (95% CI: 7.50%, 22.10%) (Figure 2A), which was substantially lower than that of SLNB alone. Compared with post-NAT US combined with SLNB, CEUS combined with SLNB showed a modestly lower observed FNR (Figure 2B). Although CEUS combined with SLNB showed the lowest observed FNR, the difference from US combined with SLNB was not statistically significant (McNemar χ^2^ = 0.571, *p*-value = 0.450). The FPR of post-NAT CEUS combined with SLNB was 22.31% (95% CI: 16.00%, 30.20%), which was higher than that of post-NAT US combined with SLNB (Figure 2C). The complete diagnostic performance of SLNB alone, US combined with SLNB, and CEUS combined with SLNB, including sensitivity, specificity, PPV, NPV, accuracy, FNR, and FPR, is summarized in Table S1.

### 3.4. False-Negative LNs Versus True-Negative LNs

**Figure 3:**
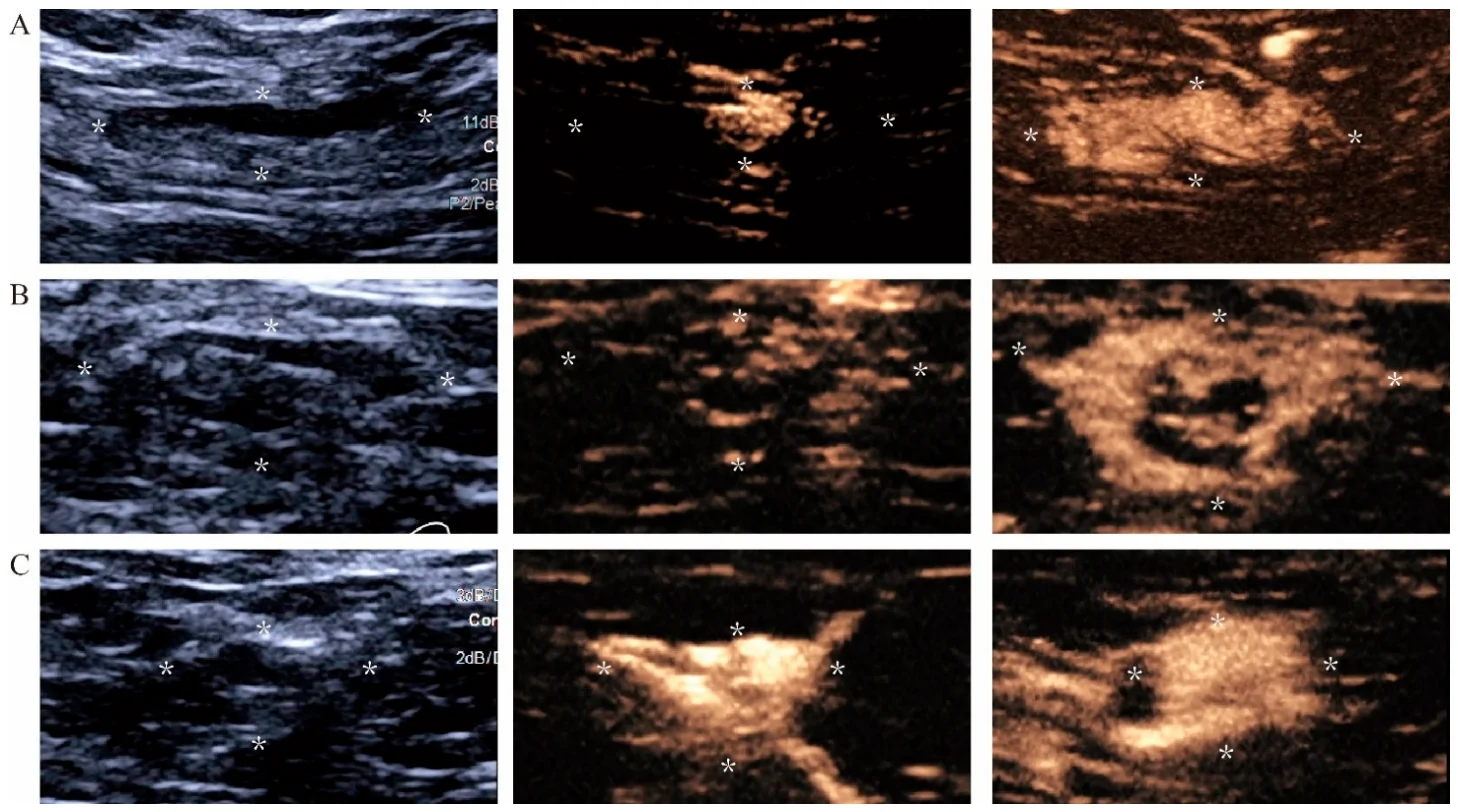
Comparison of SLN images among patients with CEUS-negative/SLNB-negative findings (**A**), CEUS-negative/SLNB-positive findings (**B**) and true-negative status (**C**). Left: B-mode ultrasonography; Middle: PCEUS; Right: ICEUS. SLN, sentinel lymph node; CEUS, contrast-enhanced ultrasound; SLNB, sentinel lymph node biopsy.

To further characterize false-negative (FN) LNs, we summarized the CEUS imaging features and clinicopathologic characteristics of the 11 FN LNs, including age, molecular subtype, and so on, and compared them with those of 101 TN LNs. In addition, because a major advantage of CEUS in axillary evaluation is its ability to localize and characterize SLNs, we separately analyzed 32 SLNs that were CEUS-negative but positive on SLNB (Figure 3). At the LN level, comparison between FN LNs (CEUS-negative/SLNB-negative) and TN LNs demonstrated that ER-positive status and HER2-negative status before NAT, residual invasive carcinoma after NAT, and malignant findings on PCEUS were associated with an increased likelihood of residual metastatic nodal disease Table S2). In contrast, comparison between misdiagnosed LNs (CEUS-negative/SLNB-positive) and TN LNs revealed that residual SLN metastasis was more frequently observed in patients with ER-positive, PR-positive, and HER2-negative tumors before NAT, and residual invasive carcinoma after NAT (Table S3).

### 3.5. Univariable and Multivariable Analyses of LN Pathological Status

**Figure 4:**
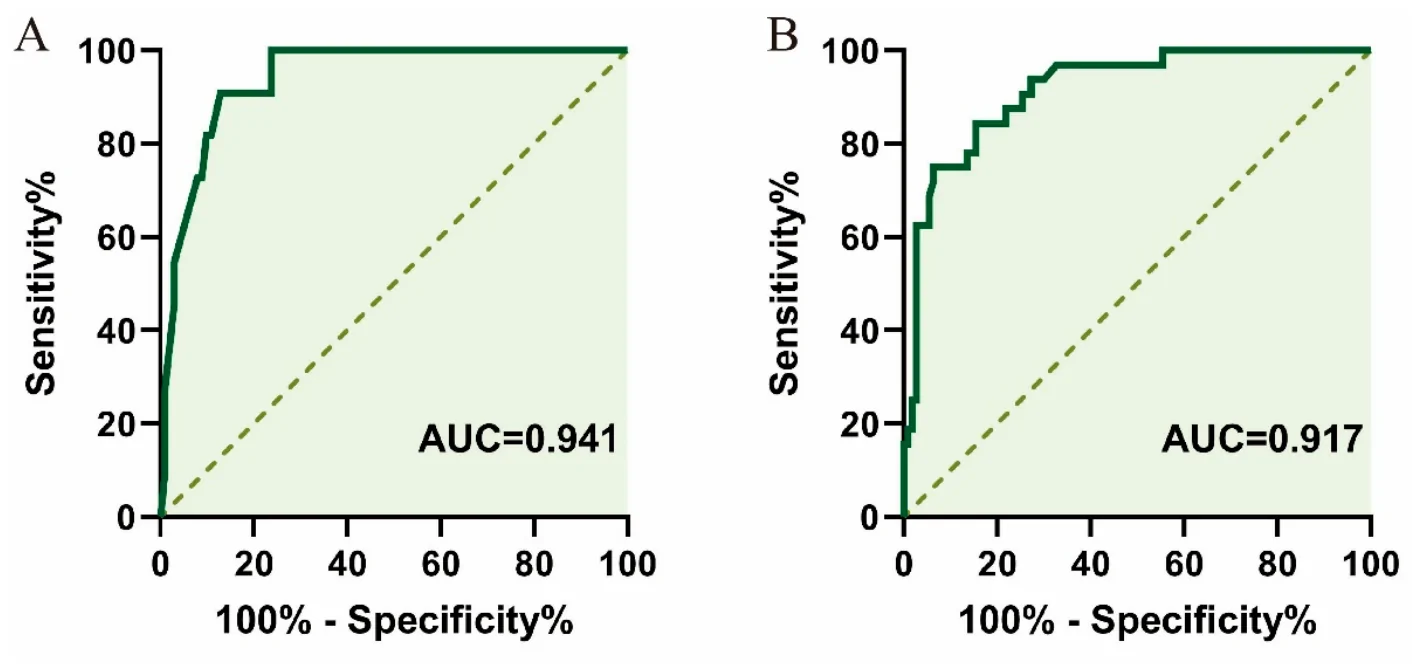
ROC curves from multivariate GEE regression analyses. (**A**) ROC curve comparing CEUS-negative/SLNB-negative LNs with TN LNs (*n* = 112; events = 11); (**B**) ROC curve comparing CEUS-negative/SLNB-positive LNs with TN LNs (*n* = 133; events = 32). The dashed line represents the reference line for no discrimination (AUC = 0.5). ROC, receiver operating characteristic; LN, lymph node; CEUS, contrast-enhanced ultrasound; SLNB, sentinel lymph node biopsy; TN, true-negative.

To better characterize factors associated with LN outcomes, univariable generalized estimating equation (GEE) analyses were performed for the three LN groups separately. Comparison between the CEUS-negative/SLNB-negative LNs (SLNs = 11) and TN LNs (SLNs = 101) groups demonstrated that ER positivity, HER2 negativity before NAT, larger primary tumor size, residual invasive carcinoma after NAT, and malignant findings on PCEUS were associated with higher odds of FN LN status (Table 2). Among these variables, HER2 positivity before NAT was associated with the lowest odds of FN LN status (OR, 0.07; 95% CI: 0.02, 0.37), whereas residual invasive carcinoma after NAT was associated with the highest odds (OR, 18.08; 95% CI: 2.15, 152.33). Univariable GEE analysis comparing the CEUS-negative/SLNB-positive LNs (SLNs = 32) and TN LNs (SLNs = 101) groups yielded similar findings (Table 2). CEUS-negative but SLNB-positive status was associated with ER positivity, PR positivity, HER2 negativity before NAT, and residual invasive carcinoma after NAT. Consistent with the findings in the CEUS-negative/SLNB-negative group, HER2 positivity before NAT was associated with lower odds of CEUS-negative/SLNB-positive status (OR, 0.12; 95% CI: 0.05, 0.30), whereas residual invasive carcinoma after NAT was associated with higher odds (OR, 31.02; 95% CI: 6.78, 141.90). LASSO logistic regression was used for exploratory selection of candidate indicator terms. The cross-validation (CV) curve of LASSO was presented in Figure S2. Candidate terms with nonzero coefficients at the selected penalty parameter were subsequently entered into the multivariable GEE models (Table 3). In the comparison between the CEUS-negative/SLNB-negative LNs and TN LNs, HER2 positivity before NAT remained independently associated with lower odds of FN LN status in the multivariable GEE model (OR, 0.09; 95% CI: 0.01, 0.88). The corresponding exploratory model showed high apparent discrimination within the study dataset, yielding an AUC of 0.941 (CI: 0.890, 0.991) for differentiating CEUS-negative/SLNB-negative from TN LN outcomes (Figure 4A). In the comparison between the CEUS-negative/SLNB-positive and TN LNs, ER positivity before NAT (OR, 7.30; 95% CI: 1.14, 46.73) and residual invasive carcinoma after NAT (OR, 23.80; 95% CI: 5.85, 96.84) remained independently associated with higher odds of pathologically positive SLNs despite negative CEUS findings. The corresponding exploratory GEE model demonstrated high apparent discrimination within the study dataset, with an AUC of 0.917 (CI: 0.866, 0.968), with ER positivity before NAT and residual invasive carcinoma after NAT independently associated with CEUS-negative/SLNB-positive status (Figure 4B).

## 4. Discussion

In this retrospective cohort, CEUS combined with SLNB showed a numerically lower observed FNR than both SLNB alone and post-NAT US combined with SLNB. However, the difference between CEUS combined with SLNB and US combined with SLNB did not reach statistical significance, indicating that the incremental diagnostic benefit of CEUS over conventional US remains uncertain. The relatively small number of false-negative cases may have limited the statistical power to detect differences between the two approaches. Nevertheless, the lower observed FNR with CEUS provides a rationale for further investigation of its potential complementary role in post-NAT axillary assessment. The potential advantage of CEUS likely reflects its ability to visualize lymphatic drainage, nodal perfusion, and microvascular features that are not readily characterized by conventional ultrasonography. In addition, its real-time localization and tracking capability may facilitate more accurate characterization of sentinel and non-sentinel LNs. Despite these advantages, deeply located or micrometastatic LNs, which can only be identified at surgical dissection, may remain undetected on CEUS, contributing to FN findings [24,25]. Comparison between FN and TN LNs demonstrated that ER positivity and HER2 negativity before NAT, breast cancer size and residual invasive carcinoma after NAT, and suspicious PCEUS findings were associated with residual nodal involvement. These findings may help identify patients who warrant particular attention during post-NAT axillary assessment. A subset of SLNs demonstrated benign CEUS findings but were pathologically positive on SLNB. Although not classified as conventional FN LNs, these CEUS-misclassified SLNs were also associated with ER positivity, PR positivity, HER2 negativity before NAT, and residual invasive carcinoma after NAT. These findings indicate that negative CEUS findings should not be interpreted as definitive evidence of the absence of metastatic SLN involvement, particularly in patients with these biologic characteristics. However, prospective studies are needed to determine whether these factors, alone or in combination with CEUS findings, can provide clinically useful risk stratification for axillary management.

Although a previous meta-analysis reported an FNR ranging from 5.1% to 43% for post-NAT SLNB [26], the relatively high FNR of 30.12% observed with SLNB alone in the present study remained relatively high. This may be attributable to treatment-related fibrosis and heterogeneous nodal response after NAT, which impair the accuracy of SLN identification [27,28]. In addition, selection or spectrum bias in real-world clinical practice may have contributed, as patients with obvious residual axillary disease are less likely to undergo CEUS evaluation, which is more costly and procedurally complex than conventional B-mode ultrasonography. Consequently, our cohort may better reflect a real-world population with more challenging disease characteristics, underscoring the need for individualized axillary assessment strategies. However, SLNB remains an important axillary staging approach after NAT, and its accuracy can be improved by optimized techniques such as dual-tracer mapping and retrieval of ≥3 SLNs [29]. Furthermore, retrieval of the clipped node may further reduce the risk of false-negative SLNB because the clipped node may not correspond to an SLN after NAT [29]. Targeted axillary dissection (TAD), combining SLNB with targeted removal of the previously biopsied and clipped node, was subsequently developed to further reduce the risk of false-negative axillary staging and has shown its potential to facilitate omission of ALND [29,30,31,32,33]. However, TAD requires clip placement and additional localization procedures, which may increase procedural complexity and cost and may limit its accessibility in some settings. In contrast, US and CEUS are nonradioactive, real-time, and widely available imaging techniques that may provide complementary information for axillary staging. Conventional axillary US can assist post-NAT axillary assessment, although its ability to exclude residual nodal disease remains limited [34]. CEUS may provide additional information on nodal perfusion and enhancement patterns and therefore represents a potential complementary imaging approach [35]. However, because optimized SLNB and TAD were not systematically evaluated in our cohort, whether CEUS provides additional benefit beyond these established approaches remains uncertain and should be investigated prospectively. The FPR associated with CEUS combined with SLNB is slightly higher than that observed with conventional ultrasound plus SLNB. One possible explanation is that CEUS may identify LNs not readily detectable on B-mode imaging, and LNs frequently exhibit fibrotic remodeling after NAT. Importantly, because the CEUS features of fibrosis and malignant infiltration are sonographically similar, the probability of false-positive diagnoses is elevated. A higher FPR means that more patients may be flagged as having suspicious axillary nodes on preoperative CEUS; however, subsequent pathological evaluation by SLNB can distinguish benign fibrotic nodes from residual nodal metastases and thereby mitigate the clinical impact of false-positive CEUS findings. From a clinical perspective, the lower observed FNR may offer greater protection against missed residual nodal disease, whereas the modest increase in FPR does not necessarily translate into unnecessary completion ALND because sentinel-node histopathology remains the definitive reference standard. In patients at higher risk of missed micrometastatic disease, the potential benefit of reducing false-negative imaging findings may outweigh the modest increase in false-positive findings. Future studies should refine CEUS nodal enhancement criteria, potentially leveraging artificial intelligence (AI) to characterize dynamic enhancement patterns and quantitative CEUS features, with the aim of better distinguishing treatment-related fibrosis from viable tumor and potentially reducing the FPR of CEUS-assisted SLNB. Among the evaluated variables, pre-NAT HER2 status showed a strong association with LN outcomes. HER2 positivity was associated with negative CEUS findings and pathologically TN LNs, which likely reflect a greater likelihood of nodal response to NAT. This observation may be related to the enhanced treatment sensitivity of HER2-positive status. HER2-positive breast cancer exhibits relatively high rates of pCR after NAT [36,37,38,39]. Furthermore, axillary pCR rates are reportedly higher in HER2 3+ tumors than in HER2 2+/fluorescence [40,41]. Other investigations have shown that pCR after NAT correlates with favorable event-free survival outcomes in stage I–III HER2-positive breast cancer, especially in HR-negative/HER2-positive status [42,43]. These findings indicate that tumor biology may substantially influence imaging reliability after NAT. Similarly, ER negativity has been identified as an independent predictor of pCR [38], whereas the HR-positive/HER2-negative subtype is frequently associated with residual nodal disease after NAT [44]. These observations may explain why ER status before NAT demonstrated significant associations with LN outcomes in this study. Residual invasive carcinoma within the primary breast lesion was also associated with CEUS-negative/SLNB-positive SLNs in our exploratory analysis. Patients achieving breast pCR after NAT are substantially more likely to achieve ypN0 status than patients without breast pCR [45]. In addition, patients without breast pCR remain at increased risk for local and axillary recurrence after NAT [46]. Although direct histopathologic assessment of the primary tumor is not possible during preoperative CEUS evaluation of ALNs, CEUS has demonstrated promising performance in predicting breast pCR after NAT [47]. Because not all patients in this study underwent preoperative breast CEUS, breast CEUS findings were not included in the current analysis. Future studies integrating CEUS assessment of both the breast and axilla may provide additional information for assessing the risk of residual nodal disease. Moreover, smaller residual breast tumor size after NAT has been associated with axillary pCR [48,49]. Although post-NAT tumor size was significant only in univariable analysis, its association with residual axillary disease warrants further investigation, particularly in relation to residual tumor burden and post-treatment imaging assessment.

The development of noninvasive approaches to reduce FNR and improve diagnostic accuracy remains a major focus in axillary staging. Although the US cannot currently replace pathological assessment, its integration with tissue sampling and advanced imaging techniques may improve the accuracy of preoperative nodal evaluation [50]. Future development of CEUS may facilitate integrated assessment of breast lesions, ALNs, and upper-extremity lymphatic pathways, particularly in patients undergoing NAT, in whom treatment-related inflammatory changes may disrupt normal lymphatic architecture [51]. Beyond conventional evaluation of perfusion and lymphatic drainage, CEUS may increasingly serve as a functional imaging biomarker within multiparametric and response-adaptive imaging paradigms. Beyond imaging, electrophoretic and dielectrophoretic assays have been explored as noninvasive approaches for characterizing metastatic cancer cell phenotypes and may warrant further investigation as potential complementary tools for assessing residual disease after NAT [52]. Recent advances in characterization of the tumor microenvironment, including immune modulation and treatment resistance after NAT, further highlight the biologic heterogeneity of residual nodal disease at the genomic and proteomic levels [53,54,55,56,57]. Concurrently, AI-driven models integrating imaging and molecular data may improve the prediction of LN status and response to therapy before surgery [58,59,60,61]. Future precision imaging strategies will likely combine imaging biomarkers, molecular characteristics, and AI-assisted analysis to guide individualized surgical and systemic management.

Although CEUS showed a numerically lower FNR, the FNR remained above the clinically accepted threshold of 10%. This supports the role of CEUS as an adjunctive imaging tool rather than a replacement for standard axillary staging strategies and highlights the need for prospective validation. Residual false-negative findings may reflect micrometastatic disease, treatment-related alterations in lymphatic drainage, heterogeneous response to NAT, and the limited spatial resolution of CEUS for small-volume nodal disease. This study also had several limitations, including its retrospective design, single-center setting, and relatively small sample size. The retrospective design and case exclusions may have introduced selection and spectrum biases, particularly because technically unsuccessful CEUS examinations were excluded. Therefore, the findings may be most applicable to patients in whom SLNs can be successfully identified and evaluated by CEUS. Although the ROC curve demonstrates good apparent discrimination, these exploratory multivariable analyses were based on a limited number of outcome events and lacked external validation. The observed model performance should therefore be interpreted cautiously, and prospective multicenter studies are warranted to further evaluate the identified factors and their potential clinical relevance in individualized axillary management.

## 5. Conclusions

Our results demonstrate that post-NAT CEUS combined with SLNB was associated with a lower observed FNR than SLNB alone or conventional US combined with SLNB. Selected clinicopathologic characteristics were also associated with residual nodal disease and CEUS–pathology discordance. These findings suggest that CEUS may serve as a complementary tool for post-NAT axillary assessment. Prospective multicenter studies with larger cohorts and external validation are warranted before these findings can be translated into clinical practice.

## Figures and Tables

**Table 1:** Clinicopathologic Characteristics of CEUS-Negative Versus CEUS-Positive LNs.

Characteristics	CEUS Negative (SLNs = 144)	CEUS Positive (SLNs = 69)	*p* Value
Age	48.88 ± 10.25	50.29 ± 9.33	0.335
Age group (year)	0.273		
≦40	34 (23.6%)	10 (14.5%)	
40–50	37 (25.7%)	22 (31.9%)	
≧50	73 (50.7%)	37 (53.6%)	
Menopausal status	0.449		
Pre-menopause	81 (56.3%)	35 (50.7%)	
Post-menopause	63 (43.8%)	34 (49.3%)	
Before NAT			
Breast cancer size (cm)	3.45 (2.50, 5.00)	3.60 (2.35, 5.30)	0.833
Clinical T stage	0.391		
T1	9 (6.3%)	8 (11.6%)	
T2	100 (69.4%)	43 (62.3%)	
T3	33 (22.9%)	18 (26.1%)	
T4	2 (1.4%)	0 (0%)	
Clinical N stage	0.176		
N1	102 (70.8%)	41 (59.4%)	
N2	28 (19.4%)	16 (23.2%)	
N3	14 (9.7%)	12 (17.4%)	
Tumor histology	0.422		
IC	21 (14.6%)	12 (17.4%)	
IDC	113 (78.5%)	55 (79.7%)	
ILC	4 (2.8%)	2 (2.9%)	
Other	6 (4.2%)	0 (0%)	
ER	0.194		
Negative	70 (48.6%)	27 (39.1%)	
Positive	74 (51.4%)	42 (60.9%)	
PR	0.287		
Negative	78 (54.2%)	32 (46.4%)	
Positive	66 (45.8%)	37 (53.6%)	
Ki-67	0.602		
Low	36 (25.0%)	15 (21.7%)	
High	108 (75.0%)	54 (78.3%)	
HER2	0.001 *		
Negative	58 (40.3%)	45 (65.2%)	
Positive	86 (59.7%)	24 (34.8%)	
After NAT			
Breast cancer size (cm)	1.40 (0.70, 2.18)	1.60 (0.95, 2.75)	0.150
Pathological response	0.059		
Pathological complete response	61 (42.4%)	19 (27.5%)	
Residual DCIS only	14 (9.7%)	5 (7.2%)	
Residual invasive disease	69 (47.9%)	45 (65.2%)	
Type of surgery	0.482		
Mastectomy	91 (63.2%)	47 (68.1%)	
Conserving surgery	53 (36.8%)	22 (36.8%)	
The number of SLNs removed	2.00 (1.25, 4.00)	3.00 (1.00, 4.00)	0.719
The number of ALNs removed	16.00 (11.00, 21.75)	17.00 (12.00, 24.00)	0.224

* *p* < 0.05. CEUS, contrast-enhanced ultrasound; LN, lymph node.

**Table 2:** Univariate GEE analysis comparing CEUS-negative/SLNB-negative LNs with TN LNs (Left) and CEUS-negative/SLNB-positive LNs with TN LNs (Right), with analyses performed at the lymph-node level.

Variable	CEUS-/SLNB- LNs vs. TN LNs		CEUS-/SLNB+ LNs vs. TN LNs	
	*p* Value	OR (95% CI)	*p* Value	OR (95% CI)
Age	0.300	0.97 (0.92, 1.03)	0.628	1.01 (0.97, 1.05)
Age group (year)				
≦40	re	re	re	re
40–50	0.364	2.09 (0.43, 10.19)	0.093	3.57 (0.81, 15.73)
≧50	0.459	0.53 (0.10, 2.86)	0.091	3.14 (0.83, 11.79)
Menopausal status	0.255	0.44 (0.11, 1.80)	0.887	0.94 (0.41, 2.18)
Before NAT				
Breast cancer size (cm)	0.933	1.02 (0.72, 1.44)	0.440	0.89 (0.66, 1.20)
Clinical T stage				
T1	re	re	re	re
T2	/	/	/	/
T3	/	/	/	/
T4	/	/	/	/
Clinical N stage				
N1	re	re	re	re
N2	0.362	0.37 (0.04, 3.15)	0.500	1.43 (0.51, 4.05)
N3	/	/	0.792	1.21 (0.29, 5.05)
Tumor histology				
IC	re	re	re	re
IDC	/	/	0.958	0.97 (0.31, 2.99)
ILC	/	/	/	/
Other	/	/	0.788	0.72 (0.06, 8.04)
ER	0.011 *	15.54 (1.89, 127.86)	0.001 *	4.58 (1.81, 11.56)
PR	0.124	2.81 (0.75, 10.45)	0.032 *	2.55 (1.08, 6.02)
Ki-67	0.070	0.30 (0.08, 1.11)	0.086	0.45 (0.18, 1.12)
HER2	0.002 *	0.07 (0.02, 0.37)	<0.001 *	0.12 (0.05, 0.30)
After NAT				
Breast cancer size (cm)	0.050 *	1.53 (1.00, 2.35)	0.170	1.24 (0.91, 1.67)
Pathological response				
Pathological complete response	re	re	re	re
Residual DCIS only	0.305	4.45 (0.26, 77.18)	/	/
Residual invasive disease	0.008 *	18.08 (2.15,152.33)	<0.001 *	31.02 (6.78, 141.90)
P-CEUS mode	0.007 *	7.43 (1.74, 31.81)	0.412	1.73 (0.47, 6.39)
I-CEUS mode	0.460	2.45 (0.23, 26.54)	0.528	1.81 (0.29, 11.46)

* *p* < 0.05. The symbol “/” indicates statistically unstable effect estimates, defined as odds ratios < 0.0001 or >10,000 or an infinite upper 95% confidence limit. LN, lymph node; CEUS, contrast-enhanced ultrasound; SLNB, sentinel lymph node biopsy; TN, true-negative; OR, odds ratio.

**Table 3:** Multivariate GEE analysis comparing CEUS-negative/SLNB-negative LNs with TN LNs (Left) and CEUS-negative/SLNB-positive LNs with TN LNs (Right), with analyses performed at the lymph-node level.

Variable	CEUS-/SLNB- LNs vs. TN LNs		Variable	CEUS-/SLNB+ LNs vs. TN LNs	
	*p* Value	Adjusted OR (95% CI)		*p* Value	Adjusted OR (95% CI)
Before NAT			Age group (year)		
			≦40	re	re
ER	0.207	4.17 (0.46, 38.29)	40–50	0.153	4.79 (0.56, 40.94)
HER2	0.039 *	0.09 (0.01, 0.88)	≧50	0.066	9.33 (0.86, 101.09)
Ki67	0.334	0.30 (0.03, 3.46)	Before NAT		
After NAT			Breast cancer size (cm)	0.103	0.68 (0.43, 1.08)
			ER	0.036 *	7.30 (1.14, 46.73)
Pathological response			HER2	0.106	0.36 (0.11, 1.24)
			After NAT		
Pathological complete response	re	re	Pathological response		
Residual DCIS only	0.063	11.93 (0.87, 163.29)	Pathological complete response	re	re
Residual invasive disease	0.157	5.07 (0.54, 47.96)	Residual DCIS only	/	/
P-CEUS mode	0.169	7.48 (0.42, 131.95)	Residual invasive disease	<0.001 *	23.80 (5.85, 96.84)

* *p* < 0.05. The symbol “/” indicates statistically unstable effect estimates, defined as odds ratios < 0.0001 or >10,000 or an infinite upper 95% confidence limit. LN, lymph node; CEUS, contrast-enhanced ultrasound; SLNB, sentinel lymph node biopsy; TN, true-negative; OR, odds ratio.

## Data Availability

All data generated or analyzed during this study are included in this article.

## Supplementary Materials

The following supporting information can be downloaded at: https://www.mdpi.com/article/10.3390/cancers18172744/s1, Figure S1: FNR of patients. A, Patients undergoing SLNB alone (SLNs = 25); B, Patients undergoing SLNB based on post-NAT US findings (SLNs = 14). Figure S2: CV curves for LASSO logistic regression. A. CV curve comparing CEUS-negative/SLNB-negative LNs with TN LNs; B. CV curve comparing CEUS-negative/SLNB-positive LNs with TN LNs. Table S1: Diagnostic performance of SLNB, US + SLNB, and CEUS + SLNB. Table S2: Comparison of clinicopathological characteristics between CEUS-negative/SLNB-negative LNs and TN LNs. Table S3: Comparison of clinicopathological characteristics between CEUS-negative/SLNB-positive LNs and TN LNs.
