# Supplementary material for: Can Contrast-Enhanced Ultrasound Reduce the False-Negative Rate of Metastatic Axillary Lymph Nodes After Neoadjuvant Therapy in Breast Cancer?
Source: Cancers (Basel). 2026 Aug 24;18(17):2744. doi: 10.3390/cancers18172744 (PMC13564639; doi:10.3390/cancers18172744)
Supplement: Supplementary file 1 [file cancers-18-02744-s001.zip › cancers-4510093-supplementary.pdf]

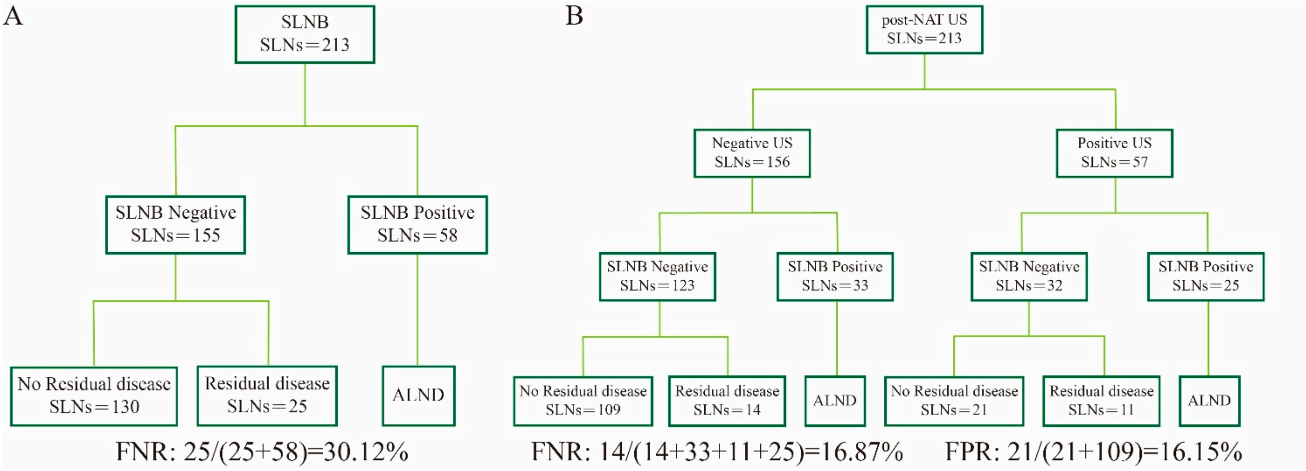

Figure S1 FNR of patients. A, Patients undergoing SLNB alone (SLNs=25); B, Patients undergoing SLNB based on post-NAT US findings (SLNs=14). FNR, false-negative rate; NAT, neoadjuvant therapy; US, ultrasound; ALND, axillary lymph node dissection; SLNB, sentinel lymph node biopsy.

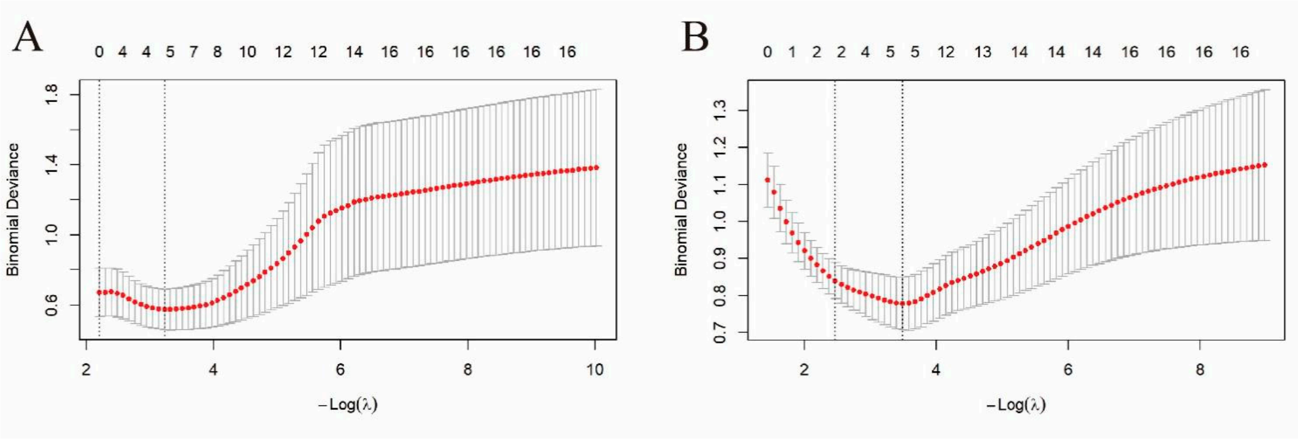

Figure S2 CV curves for LASSO logistic regression. A.CV curve comparing CEUS-negative/SLNB-negative LNs with TN LNs; B. CV curve comparing CEUS-negative/SLNB-positive LNs with TN LNs. The dashed vertical lines indicate λmin and λ1se, respectively. λmin was selected for subsequent variable selection. CV, Cross-validation.

Table S1 Diagnostic performance of SLNB, US + SLNB, and CEUS + SLNB.

Parameter	SLNB alone	US + SLNB	CEUS + SLNB
Sensitivity	69.88% (59.31-78.69%)	83.13% (73.66-89.68%)	86.75% (77.81-92.44%)
Specificity	100.00% (97.13-100.00%)	83.85% (76.56-89.18%)	77.79% (69.80-84.00%)
PPV	100.00% (93.79-100.00%)	76.67% (66.95-84.20%)	71.29% (61.81-79.20%)
NPV	83.87% (77.27-88.83%)	88.62% (81.80-93.10%)	90.18% (83.27-94.43%)
Accuracy	88.26% (83.25-91.92%)	83.57% (78.01-87.94%)	81.22% (75.44-85.90%)
FNR	30.12% (21.31-40.69%)	16.87% (10.32-26.34%)	13.25% (7.56-22.19%)
FPR	0.00% (0.00-2.87%)	16.15% (10.82-23.44%)	22.31% (16.00-30.20%)

Diagnostic performance measures are presented as estimates with corresponding 95% CIs.

Table S2 Comparison of clinicopathological characteristics between CEUS-negative/SLNB-negative LNs and TN LNs.

Characteristics	CEUS-/SLNB- LNs (SLNs=11)	TN LNs (SLNs=101)	p Value
Age	42.00 (40.00, 58.00)	49.00 (39.00, 56.00)	0.400
Age group(year)			0.227
≦40	3 (27.3%)	28 (27.7%)	
40–50	5 (45.5%)	23 (22.8%)	
≧50	3 (27.3%)	50 (49.5%)	
Menopausal status			0.436
Pre-menopause	8 (72.7%)	56 (55.4%)	
Post-menopause	3 (27.3%)	45 (44.6%)	
Before NAT			
Breast cancer size (cm)	3.40 (2.60, 5.10)	3.60 (2.50, 5.00)	0.814
Clinical T stage			0.018*
T1	0 (0.0%)	5 (5.0%)	re
T2	7 (63.6%)	72 (71.3%)	1.000
T3	2 (18.2%)	24 (23.8%)	1.000
T4	2 (18.2%)	0 (0.0%)	0.048
Clinical N stage			0.493
N1	10 (90.0%)	71 (70.3%)	
N2	1 (9.1%)	20 (19.8%)	
N3	0 (0.0%)	10 (9.9%)	
Tumor histology			0.080
IC	0 (0.0%)	16 (15.8%)	
IDC	10 (90.0%)	80 (79.2%)	
ILC	1 (9.1%)	0 (0.0%)	
Other	0 (0.0%)	5 (5.0%)	
ER			0.002*
Negative	1 (9.1%)	61 (60.4%)	
Positive	10 (90.9%)	40 (39.6%)	
PR			0.201
Negative	4 (36.4%)	62 (61.4%)	
Positive	7 (63.6%)	39 (38.6%)	
Ki-67			0.052
Low	5 (45.5%)	20 (19.8%)	
High	6 (54.5%)	81 (80.2%)	
HER2			< 0.001*
Negative	9 (81.8%)	25 (24.8%)	
Positive	2 (18.2%)	76 (75.2%)	
After NAT			
Breast cancer size (cm)	1.90 (1.10, 3.40)	1.20 (0.30, 2.10)	0.055
Pathological response			0.001*
Pathological complete response	1 (9.1%)	58 (57.4%)	re

Residual DCIS only	1 (9.1%)	13 (12.9%)	0.262
Residual invasive disease	9 (81.8%)	30 (29.7%)	0.001*
P-CEUS mode			0.010*
Benign	7 (63.6%)	94 (93.1%)	
Malignant	4 (36.4%)	7 (6.9%)	
I-CEUS mode			0.410
Benign	10 (90.0%)	94 (93.1%)	
Malignant	1 (9.1%)	7 (6.9%)	

LN, lymph node; CEUS, contrast-enhanced ultrasound; SLNB, sentinel lymph node biopsy; TN, true-negative.

Table S3 Comparison of clinicopathological characteristics between CEUS-negative/SLNB-positive LNs and TN LNs.

Characteristics	CEUS-/SLNB+ LNs (SLNs=32)	TN LNs (SLNs=101)	p Value
Age	50.56±8.34	48.70±10.83	0.375
Age group(year)			0.081
≦40	3 (9.4%)	28 (27.7%)	
40–50	9 (28.1%)	23 (22.8%)	
≧50	20 (62.5%)	50 (49.5%)	
Menopausal status			0.818
Pre-menopause	17 (53.1%)	56 (55.4%)	
Post-menopause	15 (46.9%)	45 (44.6%)	
Before NAT			
Breast cancer size (cm)	3.20 (2.43, 4.28)	3.60 (2.50, 5.00)	0.245
Clinical T stage			0.370
T1	4 (12.5%)	5 (5.0%)	
T2	21 (65.6%)	72 (71.3%)	
T3	7 (21.9%)	24 (23.8%)	
T4	0 (0.0%)	0 (0.0%)	
Clinical N stage			0.870
N1	21 (65.6%)	71 (70.3%)	
N2	7 (21.9%)	20 (19.8%)	
N3	4 (12.5%)	10 (9.9%)	
Tumor histology			0.042*
IC	5 (15.6%)	16 (15.8%)	re
IDC	23 (71.9%)	80 (79.2%)	0.883
ILC	3 (9.4%)	0 (0.0%)	0.028
Other	1 (3.1%)	5 (5.0%)	1.000
ER			< 0.001*
Negative	8 (25.0%)	61 (60.4%)	
Positive	24 (75.0%)	40 (39.6%)	
PR			0.018*
Negative	12 (37.5%)	62 (61.4%)	
Positive	20 (62.5%)	39 (38.6%)	
Ki-67			0.089
Low	11 (34.4%)	20 (19.8%)	
High	21 (65.6%)	81 (80.2%)	
HER2			< 0.001*
Negative	24 (75.0%)	25 (24.8%)	
Positive	8 (25.0%)	76 (75.2%)	
After NAT			
Breast cancer size (cm)	1.70 (1.00, 2.43)	1.20 (0.30, 2.10)	0.062
Pathological response			< 0.001*
Pathological complete response	2 (6.3%)	58 (57.4%)	re

Residual DCIS only	0 (0.0%)	13 (12.9%)	1.000
Residual invasive disease	30 (93.8%)	30 (29.7%)	< 0.001*
P-CEUS mode			0.530
Benign	28 (87.5%)	94 (93.1%)	
Malignant	4 (12.5%)	7 (6.9%)	
I-CEUS mode			0.956
Benign	30 (93.8%)	94 (93.1%)	
Malignant	2 (6.3%)	7 (6.9%)	

LN, lymph node; CEUS, contrast-enhanced ultrasound; SLNB, sentinel lymph node biopsy; TN, true-negative.
